# Modeling health risks using neural network ensembles

**DOI:** 10.1371/journal.pone.0308922

**Published:** 2024-10-09

**Authors:** Brandon M. Smith, Antonio Criminisi, Noam Sorek, Yaar Harari, Neeraj Sood, Steven B. Heymsfield

**Affiliations:** 1 Amazon.com, LLC, Washington, D. C, United States of America; 2 Amazon UK Services Ltd., London, United Kingdom; 3 Amazon.com, LLC, Israel; 4 USC Sol Price School of Public Policy, Los Angeles, CA, United States of America; 5 Pennington Biomedical Research Center, Louisiana State University System, Baton Rouge, Louisiana, United States of America; Bangor University, UNITED KINGDOM OF GREAT BRITAIN AND NORTHERN IRELAND

## Abstract

This study aims to demonstrate that demographics combined with biometrics can be used to predict obesity related chronic disease risk and produce a health risk score that outperforms body mass index (BMI)—the most commonly used biomarker for obesity. We propose training an ensemble of small neural networks to fuse demographics and biometrics inputs. The categorical outputs of the networks are then turned into a multi-dimensional risk map, which associates diverse inputs with stratified, output health risk. Our ensemble model is optimized and validated on disjoint subsets of nationally representative data (N~100,000) from the National Health and Nutrition Examination Survey (NHANES). To broaden applicability of the proposed method, we consider only non-invasive inputs that can be easily measured through modern devices. Our results show that: (a) neural networks can predict individual conditions (e.g., diabetes, hypertension) or the union of multiple (e.g., nine) health conditions; (b) Softmax model outputs can be used to stratify individual- or any-condition risk; (c) ensembles of neural networks improve generalizability; (d) multiple-input models outperform BMI (e.g., 75.1% area under the receiver operator curve for eight-input, any-condition models compared to 64.2% for BMI); (e) small neural networks are as effective as larger ones for the inference tasks considered; the proposed models are small enough that they can be expressed as human-readable equations, and they can be adapted to clinical settings to identify high-risk, undiagnosed populations.

## Introduction

Body composition is associated with cardiorespiratory fitness and longitudinal health outcomes [1,2]. Excess adiposity impairs functional performance and is a major risk factor for developing chronic diseases [3–7]. Similarly, smoking [8], excessive alcohol consumption [9], ageing [10] and sedentary behaviors [11] are all linked to adverse health outcomes. The increased risk of chronic diseases that accompany such risk factors contribute to estimated medical costs in the hundreds of billions of dollars, both in the U.S. [12,13] and abroad [14].

In clinical practice and public health, obesity-related health risk levels are defined using mainly body mass index (BMI), where adults with BMI ≥25 and ≥30 kg/m^2^ are classified as overweight and obese, respectively [15–17]. However, BMI alone cannot discern body fat from lean tissue, which often leads to risk level misclassification [18,19]. The American Medical Association (AMA) released a press release on June 14, 2023 critical of BMI: “Due to significant limitations associated with the widespread use of BMI in clinical settings, the AMA suggests that it be used in conjunction with other valid measures of risk” [20]. Polygenic risk scores such as the one developed in the GenoVA Study [21] can more accurately predict the risk of various conditions (including cancer), through laboratory-based genetic analysis. But is it possible to get accurate risk assessment with inexpensive, non-invasive, self-administered rapid tests that people can run at home by themselves and obtain results within seconds?

Our hypothesis is that using multiple demographic and non-invasive body composition biomarkers (e.g., sex, percent body fat, waist circumference, hip circumference) may achieve that goal. Intuitively, building a more complete picture of health risk from multiple measurements should be more accurate than single measurements alone (e.g., waist circumference) or pairs of measurements (e.g., height and weight in BMI). We prioritize non-invasive inputs because they reduce user experience friction and improve the feasibility of providing an improved risk assessment at scale. However, an important question remains: What is the best way to measure and combine different biomarkers together for maximum accuracy?

Due to complex correlations and interactions between different input biomarkers, a manually designed system is likely to be fragile or lack generalizability. Here we propose to use a neural network (NN) [22], a specific type of machine learning (ML) model whose parameters are automatically selected through the minimization of an objective (a.k.a. loss) function defined on a large repository of representative, labeled data. We employ NNs because they are well-studied, and form the basis for noteworthy state-of-the-art deep learning systems, such as ChatGPT (https://openai.com/blog/chatgpt) and Stable Diffusion (https://stability.ai/blog/stable-diffusion-public-release). We refer readers to Sarker [23] for a survey of other ML techniques.

Typically, the output of a NN can be categorical (e.g., cat, dog) or numerical (e.g., house prices, temperature). When the output is categorical the network is called a *classifier*; when it is numerical the network is called a *regressor*. The parameters of a NN are typically optimized on a *training* set, which contains pairs of {input data, output label}. The best set of hyperparameters (e.g., learning rate, model architecture, training loss function) are typically selected on a disjoint *validation* set, and then tested on another disjoint *test* set to measure the model’s accuracy.

**Fig 1:**
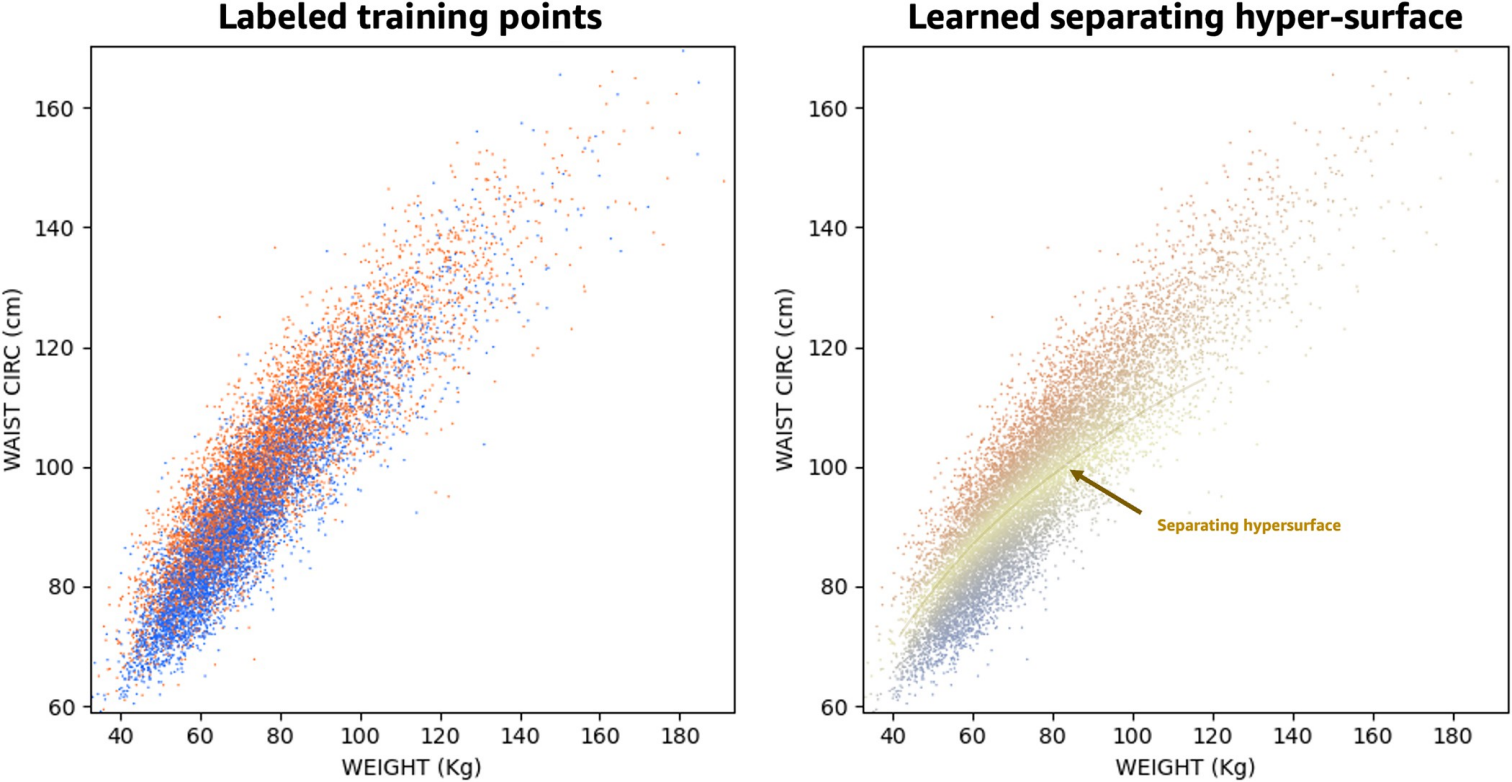
An example of training a classification network. In the left panel, each dot corresponds to weight and waist circumference measurements for thousands of participants in NHANES. The dot colors (blue/orange) correspond to participants ground truth class (negative/positive to a given health condition, e.g., hypertension). In the right panel, a neural network classifies previously unseen data points as positive or negative according to a separating hypersurface in as many dimensions as the input biomarkers.

Fig 1 shows an example of training a classification network. In the left panel, each dot corresponds to weight and waist circumference measurements for thousands of participants in NHANES. Notice how in the chosen two dimensions (weight, waist) the two classes are not cleanly separable. As in previous work [24] we employ NNs to classify previously unseen data points as positive or negative (Fig 1 right). The network implicitly defines a separating hypersurface in as many dimensions as the input biomarkers.

Recently, anthropometric measurements (e.g., ABSI [25], RFM [26]) and body composition biomarkers [27,28] were employed with a similar objective in mind. In that line of work manually designed formulae were used to combine input measurements. In this work we use NN ensembles to combine and downweight or upweight inputs. The network parameters are *optimized automatically* by minimizing a well-defined objective function (binary cross entropy) over labeled data. Automatic, backpropagation-based parameter learning [29] makes it easy to extend our methodology to many input markers (not only body composition ones) and health conditions.

The network can be made simple or complex by varying its architecture and number of tunable parameters. A tension typically exists between the accuracy and interpretability of model predictions [30], but we demonstrate that the network can be relatively simple in this case and still achieve results similar to larger networks. In fact, the proposed network architecture is so simple (one hidden layer with two nodes) that the final model can be faithfully represented by a few human-readable equations, enabling better interpretability [31] and experimentation by non-machine learning experts via a spreadsheet, for example. If more complexity is acceptable, we demonstrate that *ensembles* of NNs yield more generalizable results than single networks.

As shown later, using multiple input biomarkers leads to greater risk prediction accuracy. The flip side is that gathering multiple measurements takes time and effort, especially if invasive clinical tests are needed. Here we focus on non-invasive inputs that can be easily measured through modern devices and smartphone-based technologies such as Apple Watch (https://www.apple.com/uk/healthcare/apple-watch/), FitBit (https://healthsolutions.fitbit.com/), MeThreeSixty (https://www.methreesixty.com/), and Amazon Halo (https://www.amazon.science/latest-news/the-science-behind-the-amazon-halo-band-body-feature). Such devices make it increasingly easy to measure and track body composition [32], amount and intensity of physical activity [33], diet quality [34], heart rate and heart rate variability [35], thus making accurate health risk prediction and tracking available to all.

In summary, the aim of this paper is to propose a methodology, based on NNs applied to multiple biomarkers that can be easily measured using modern technology, that can improve upon BMI.

## Methods

### Software development

Our models were developed with Python (https://www.python.org/) and PyTorch (https://pytorch.org/). Tables and figures were generated using Matplotlib (https://matplotlib.org/), MATLAB (https://www.mathworks.com/products/matlab.html) and Microsoft Excel (https://www.office.com/).

### Data source

All analyses in this study were conducted using the National Health and Nutrition Survey (NHANES) dataset, collected between the years 1999 and 2020. The dataset was accessed on October 31, 2022 and can be downloaded for free from the CDC website: https://wwwn.cdc.gov/nchs/nhanes/. The dataset comprises a total of more than 100,000 unique participants with data related to demographics, body composition, fitness habits, eating habits and medical conditions. No authors had access to information that could identify individual participants. A consort diagram and analysis of the participants characteristics are presented in S1 Fig and S1 Table, respectively. For all experiments and evaluations, NHANES sample weights were applied to yield estimates representative of the U.S. civilian non-institutionalized population.

This study did not require IRB approval as it was deemed to be not human subjects research. The specimens or data were not collected specifically for this study and no one on the study team has access to the subject identifiers linked to the specimens or data. We made this determination using the decision tool provided by the National Institutes for Health at https://grants.nih.gov/policy/humansubjects/hs-decision.htm.

### Health conditions

This study considers the prevalence of any one of nine common health conditions: hypertension, diabetes, arthritis, coronary heart disease, angina, congestive heart failure, had a heart attack, had a stroke, and cancer (general malignancy). Several of these conditions overlap (e.g., angina and coronary heart disease). However, we ultimately combine them via their union as described in the **Condition-agnostic health risks** section, thus it is acceptable if conditions are correlated or partially redundant.

Previous work, such as López-Martínez et al. [24], Klados et al. [36], Huang and Huang [37], and Criminisi et al. [38], define being positive to a condition based solely on participants’ answers to questions (e.g., “Has a doctor ever told you that you have diabetes?”) in the NHANES survey (see S1 File). This study instead defines positive to a condition based on *either* participants’ answers (diagnosed) or based on examination measurements in NHANES (previously undiagnosed). Specifically, for hypertension, we add participants with systolic blood pressure ≥130 mm Hg or diastolic blood pressure ≥80 mm Hg; for diabetes, we add participants with fasting glucose ≥7.0 mmol/L or glycohemoglobin ≥6.5% or glucose tolerance test ≥11.1 mmol/L.

### Biomarkers (input features)

We investigate the use of the following biomarkers and demographics: BMI (baseline), percentage body fat (PBF), waist circumference, thigh circumference, hip circumference, height, weight, age, sex, and ethnicity. These input features are an intersection of measurements widely available in the NHANES dataset and measurements that are now convenient and inexpensive with modern devices and smartphones. For example, smart scales commonly provide body fat measurements, and Amazon’s Halo (https://www.amazon.science/latest-news/the-science-behind-the-amazon-halo-band-body-feature) uses computer vision to measure body composition from smartphone images. For ensembles that take more than one feature as input, we do not consider BMI. Instead, we provide height and weight as separate input options, and allow the model to *learn* an optimal combination from the data. The model can combine height and weight internally in a way that closely approximates the BMI equation, thereby making a separate BMI input redundant, or it can combine height and weight in a different, possibly better way, i.e., to generalize beyond the BMI equation. Input features are described in detail in S2 File.

### Neural networks

This study follows a typical neural network (NN) design strategy. The architecture of the NN was selected empirically. The architecture that minimizes the loss on the validation set was chosen. We considered models with 0, 1, 2, and 3 hidden layers [39], and with 2, 4, 8, 16, 32, or 64 nodes each. Each hidden layer was allowed equal or fewer nodes compared to its predecessor.

All NNs used in this study were trained using the Adam optimization algorithm [40]. For better convergence, input features were normalized, i.e., mean-centered and scaled to the unit sphere; batch normalization [41] was added between network layers to further accelerate and stabilize training. All network activation layers were sigmoid functions [39]. Binary cross-entropy [39] was used as the objective function during training. A small L1 regularization loss [39] on model weights was added (lambda = 0.01) to the overall loss to reduce overfitting.

Model parameters in all experiments were optimized using a training set (40%), model hyperparameters (e.g., learning rate, training loss function) were selected using a validation set (20%), and final model performance was assessed using a test set of previously unseen examples (40%). Train, validation, and test sets were created randomly once at the beginning of all experiments, were reused throughout, and were completely disjoint.

### Neural network ensembles

Combining different machine learning models into ensembles has consistently proved to be an effective technique for improving generalization [42,43]. Research has demonstrated that ensemble methods reduce the propensity of overfitting [44], including in the context of imbalanced data [45], and can reduce the likelihood of false-positives [46]. Ensembles can improve predictive performance because they combine diverse models that may excel in different areas, or capture different aspects of the data [47].

**Fig 2:**
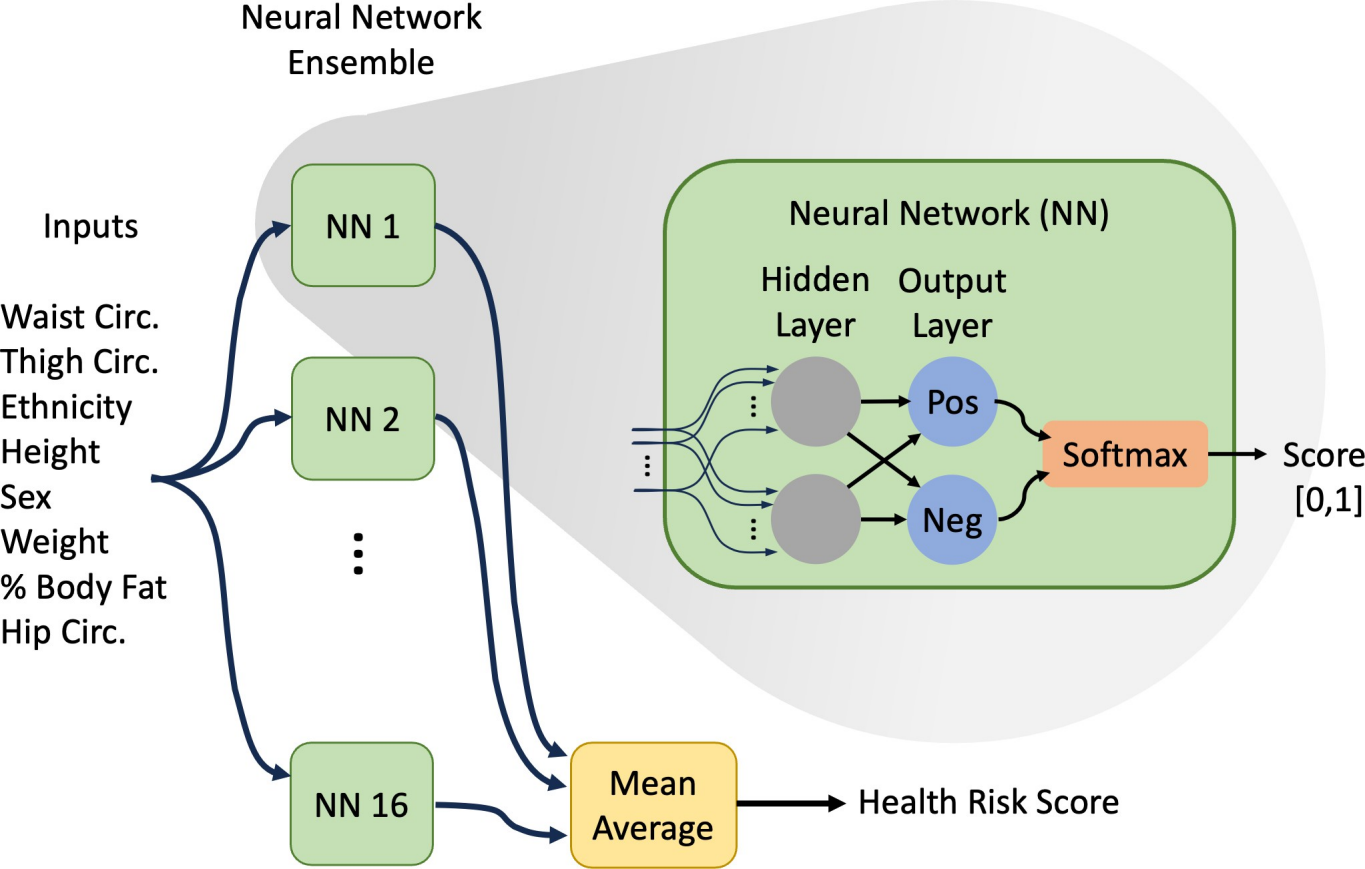
Visual overview of the proposed neural network ensemble.

In this work we build ensembles of NNs trained using randomly bagged [48] (with replacement) 50% subsets of the training data. The output of the ensemble is the average of the individual Softmax [49] outputs of each component network. A visual overview is shown in Fig 2. The number of NNs in the ensemble was selected empirically to maximize average accuracy and minimize variability (standard deviation) in accuracy between different experiment trials, while balancing increased training time, i.e., improvements become negligible beyond a certain ensemble size, as described in the Results section. All remaining results in this work were obtained with ensembles of N = 16 NNs.

### Data imputation

We improve model performance by imputing missing biomarker and demographic entries in NHANES, which effectively increases the size of dataset. For example, if we consider nine health conditions and sex, ethnicity, waist, thigh, height, weight, and PBF, data imputation allows the dataset to grow from 15,395 subjects with complete input values to 49,243 subjects. Imputation also allows us to consider thigh and hip circumference together, which were never recorded simultaneously for the same subject in NHANES. Model performance improvements are included in Table A in S3 File.

We adopt a strategy substantially similar to Xu et al. [50], described in detail in S4 File. To avoid spurious imputations, we only impute missing measurements for subjects that have other correlated input features available. For example, we only impute hip circumference measurements if other correlated measurements are available (e.g., thigh circumference or gynoid fat).

### From disease classification to risk regression

**Fig 3:**
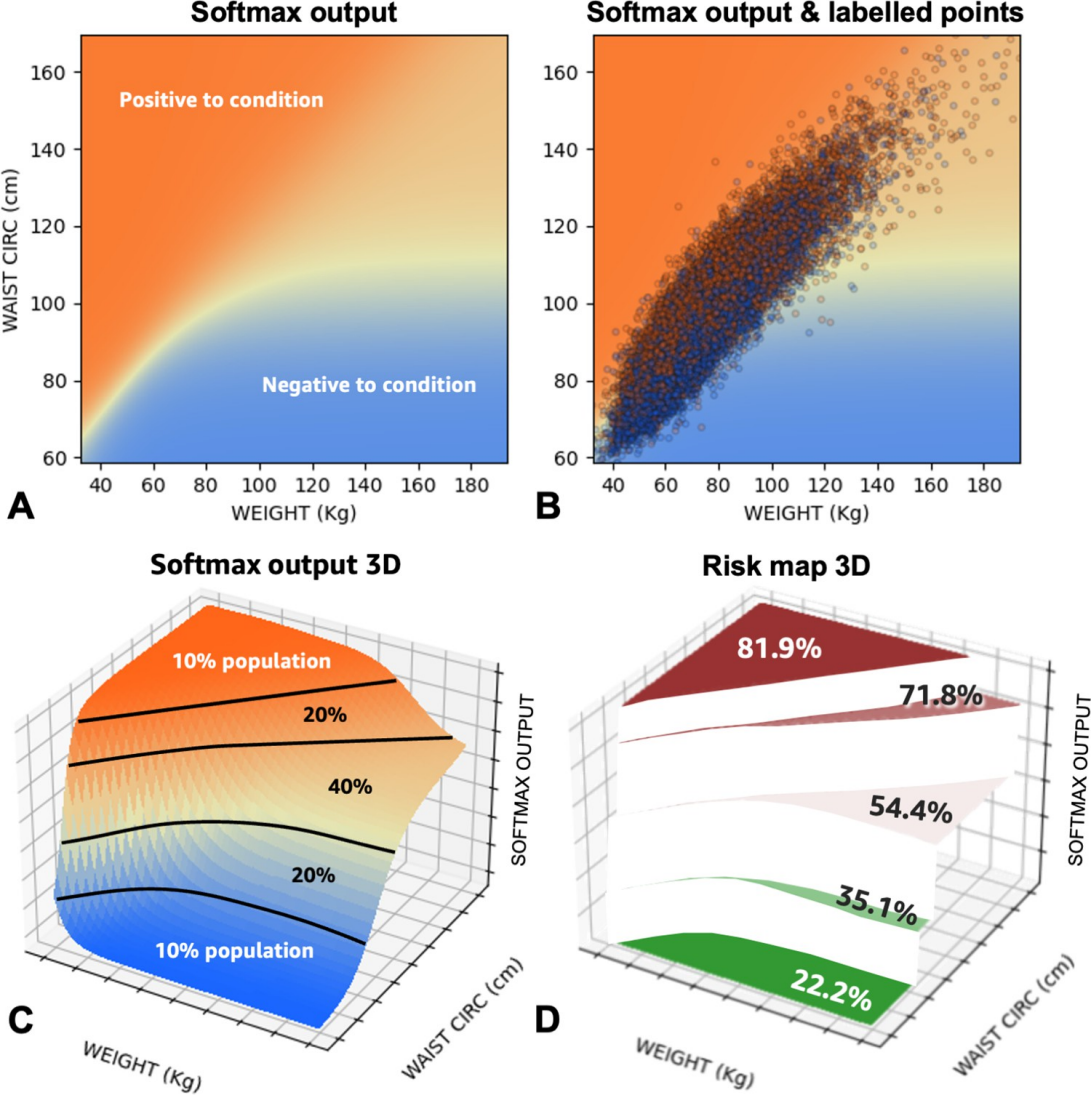
A network’s categorical output can be converted into a risk map. The Softmax network output (**A**) can be interpreted as a surface defined over the multi-dimensional space of input biomarkers (**C**). We can then slice that surface into regions based on percentiles of Softmax network output. In each of these “risk regions” we calculate the condition prevalence by simply counting how many participants in that region are positive, versus the total number of people in the region (**D**). The multi-dimensional shape of the risk regions is defined by the network output, and the actual risk associated with each region is simply the condition prevalence in that region.

**Fig 4:**
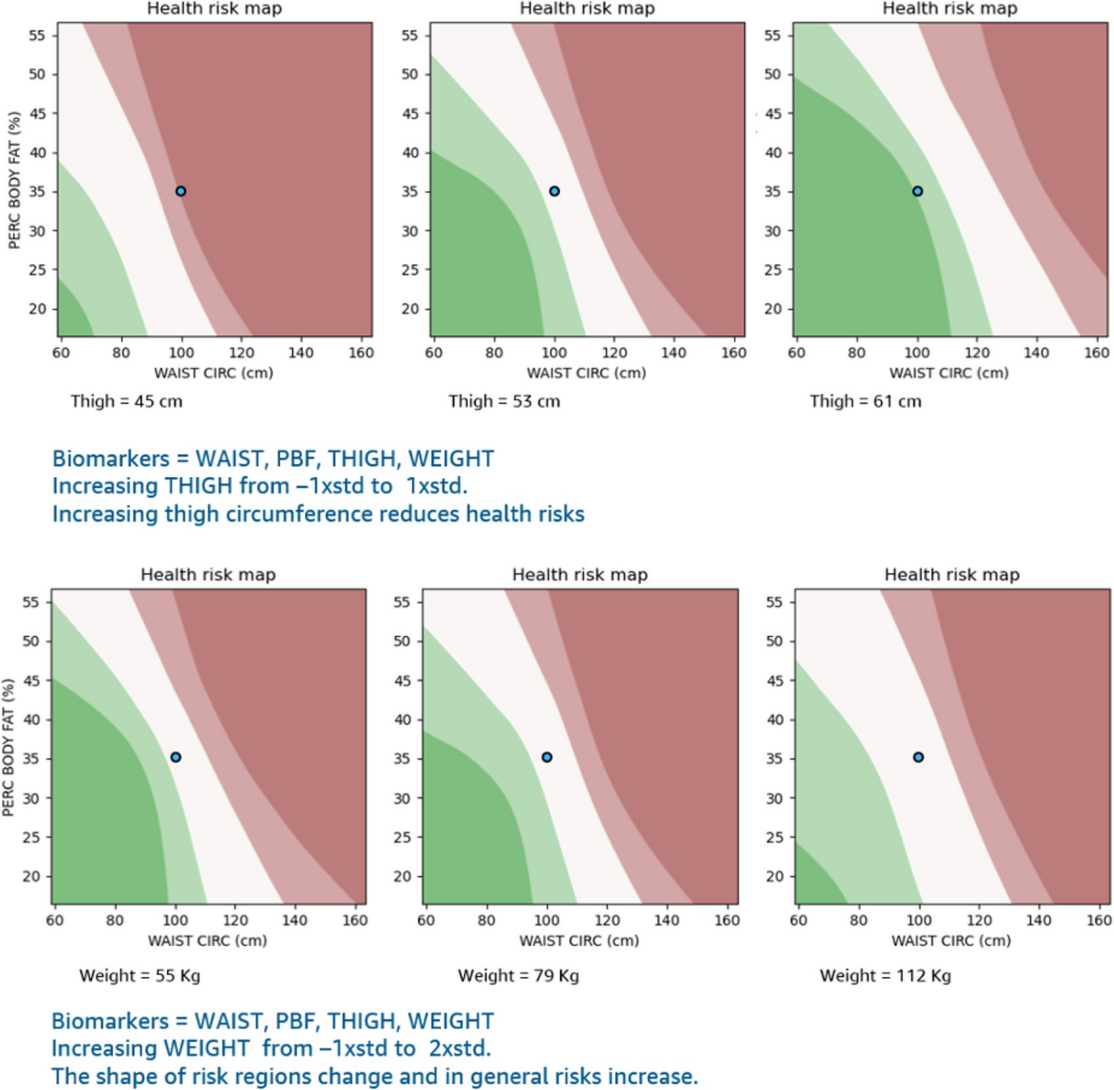
Visualization of 3D health risk maps via 2D slices for {percent body fat, waist circumference, thigh circumference} and {percent body fat, waist circumference, weight} input sets. The dark maroon regions correspond to the top 10% of Softmax model outputs; light maroon is the next 20%, white is the middle 40%, light green is the next 20%, and dark green is the lowest 10%.

In previous work, machine learning models stop at the level of classifying patients as positive or negative (to a given condition). Here, we wish to estimate health risks (a continuous, numerical output) from input biomarkers and demographics. This is a typical regression task. However, in NHANES, we do not have a ground truth risk associated with each participant; instead, we have associated categorical labels (e.g., has/does not have a medical condition). As illustrated in Fig 3, we turn the network’s categorical output into a risk map as follows. The Softmax network output (Fig 3A) can be interpreted as a surface defined over the multi-dimensional space of input features (Fig 3C). We can then slice that surface into regions based on percentiles of Softmax network output (here we use 100 to 90^th^ percentile, 90 to 70^th^ percentile, 70^th^ to 30^th^ percentile, 30^th^ to 10^th^ percentile, and below 10^th^ percentile). In each of these “risk regions” we calculate the condition prevalence by simply counting how many participants in that region are positive, versus the total number of people in the region (Fig 3D). The multi-dimensional shape of the risk regions is defined by the network output, and the actual risk associated with each region is simply the condition prevalence in that region. More or less fine-grained risk regions can be defined for the same NN essentially creating a function that maps the Softmax network output to predicted condition prevalence. Once a risk map has been computed, it can be visualized in 2D as one or more conventional medical charts (Fig 4). It can be used by practitioners without the need to run any complex algorithm; all practitioners need is the input biomarkers and demographics for their patients and the risk map would transform the input features to a predicted disease risk.

Because the models we propose are relatively small, we additionally provide explicit equations in S5 File for one model in the best-performing ensemble. This allows the model to be implemented in a spreadsheet, for example, and enables non-machine learning experts to run “what if” scenarios in real time to understand how changes in biomarkers would translate to lower or higher disease risk.

### Evaluation metrics

In this study, we want to compare different sets of input markers and different network architectures and find the combination that is most effective at predicting health risks. Binary classifiers are usually assessed through test accuracy or measures derived from the confusion matrix. However, those measures do not capture the quality of risk stratification. For a model to be good at stratifying health risks it should be able to order test cases so that negative-condition subjects are separated from positive-condition subjects. An ideal ordering would be one with maximum risk separation, i.e., one that places all negative-condition subjects before all positive-condition subjects.

Here we consider the area under the receiver operator characteristic curve (AUROC)—a rank order statistic—as our key evaluation metric. We observe that a classifier producing random outputs will yield an AUROC close to 50%, and a classifier producing Softmax outputs that order subjects ideally according to health risks will yield an AUROC of 100%.

### Condition-agnostic health risks

Previous work has used classifiers to identify whether a patient is positive/negative to a *specific* condition, *e*.*g*., diabetes [51], hypertension [24], and CVD risk [36]. Our proposed algorithm can also be used that way, which we demonstrate for diabetes and hypertension. Additionally, we are interested in creating a *condition-agnostic* or any-condition health risk, *i*.*e*., we are trying to answer the question: “Is this person likely to have a health condition or not?”, independent from the condition itself. A single health risk score is attractive in its simplicity and can have similar utility as other single-valued biometrics, such as BMI (e.g., in health insurance to determine a customer’s eligibility for lifestyle interventions), but with much lower false negative and false positive rates. To address this task here we consider nine common health conditions: hypertension, diabetes, arthritis, coronary heart disease, angina, congestive heart failure, had a heart attack, had a stroke, and cancer (general malignancy). In our ground-truth labeled dataset, a participant is deemed positive if they are positive to at least one of those conditions; and negative if they are negative to all conditions. The set and number of conditions can be changed without changing the methodology itself.

## Results and discussion

Our modeling approach yielded six main findings that are presented and discussed in the following sections.

### Small neural networks are effective for health risk prediction

For the task of health risk prediction from a few demographic and biometric values, NN models need not be large or complex. As shown in Table 1, for this task, we observe no significant benefits in adding more than a single hidden layer with two nodes (P → 2 → 2). Deeper and/or wider models do not significantly improve results in this case. We therefore use this simple architecture for subsequent experiments.

**Table 1:** Performance of different neural network architectures vs. number of input features using AUROC (test) as the evaluation metric.

Number of	Architecture	Number of Inputs, P								
Hidden Layers		1	2	3	4	5	6	7	8	
0	Linear Classifier	69.3%	69.3%	68.8%	69.3%	68.8%	69.3%	68.8%	68.8%	
1	P → 2 → 2	68.7%	72.6%	73.5%	74.4%	74.6%	74.7%	75.0%	75.1%	⟵
	P → 8 → 2	68.7%	72.6%	73.5%	74.4%	74.6%	74.7%	75.0%	75.1%	
	P → 64 → 2	68.7%	72.6%	73.5%	74.4%	74.6%	74.7%	75.0%	75.1%	
2	P → 2 → 2 → 2	68.7%	72.6%	73.5%	74.4%	74.6%	74.7%	75.0%	75.1%	
	P → 8 → 4 → 2	68.7%	72.6%	73.5%	74.4%	74.6%	74.7%	75.0%	75.1%	
	P → 64 → 32 → 2	68.7%	72.6%	73.5%	74.4%	74.6%	74.7%	75.0%	75.1%	
3	P → 2 → 2 → 2 → 2	68.4%	72.4%	73.5%	74.4%	74.6%	74.7%	75.0%	75.1%	

For each cell, we exhaustively trained and evaluated any-condition prediction ensemble models with all combinations of P biomarker inputs. Available inpute features were: Waist, thigh, and hip circumferences, ethnicity, height, sex, percent body fat, and weight. Arrows under “Architecture” indicate connections from each network layer to the next; the number of nodes in each layer is listed between arrows.

For greater than one input biomarker, one hidden layer provides sufficient nonlinearity to the NN model, and this nonlinearity produces significantly better results compared to a logistic regression baseline. Performance improves among NN architectures as the number of inputs increases. For the eight-biomarker input setting, we show in Fig A in S6 File that NN ensembles perform better than XGBoost (https://xgboost.readthedocs.io/en/stable/), a popular ML library based on gradient boosting for structured data, including medical data [52].

### Multiple biometrics considered jointly significantly outperform BMI alone for health risk prediction

Model ensembles that use multiple biomarker inputs perform much better than models that use only BMI as input for condition-specific and any-condition health risk prediction as shown in Table 2.

**Table 2:** Comparison of health risk prediction performance using BMI-only input (baseline) and multiple input features for diabetes, hypertension, and any-condition models.

Output	Prevalence		Input	AUROC		Prev. Top	Specificity	Sensitivity
Condition Type	Test	Train	Biomarkers	Test	Train	25% (Test)	(Test)	(Test)
Diabetes	12.0%	12.6%	BMI	69.0%	69.6%	21.4%	77.0%	47.3%
			Waist, Weight, Ethnicity, Thigh, Height, Sex, PBF	79.8%	79.5%	29.4%	78.3%	64.5%
Hypertension	48.7%	49.8%	BMI	64.4%	64.9%	62.9%	67.5%	53.0%
			Waist, Weight, Ethnicity, Sex, Thigh, Height	72.2%	73.3%	73.4%	71.0%	60.8%
Any	60.5%	61.5%	BMI	64.2%	64.7%	75.6%	81.5%	35.8%
			Waist, Thigh, Ethnicity, Height, Sex, Weight, PBF, Hip	75.1%	76.1%	86.6%	75.6%	60.3%

In addition to AUROC, we include specificity, sensitivity, and prevalence among the top 25% of Softmax examples (“Prev. Top 25%”) as intuitive metrics for reference.

We observe good model generalization, i.e., the drop from *AUROC Train* to *AUROC Test* is small (1% or less), and low variability for the best multi-input, any-condition ensemble model when bootstrapping [53] across 100 random initializations and test/val/train resamples: AUROC test min, max, and standard deviation (SD) were min = 83.13%, max = 84.23%, SD = 0.29%; specificity (test) were min = 80.20%, max = 1.90%], SD = 0.40%; and sensitivity (test) were min = 69.37%, max = 70.94%, SD = 0.32%. For simplicity, we show results for a single trial of each ensemble model using a single predefined train/validation/test split. For condition-specific models and any-condition models, the AUROC improvement from BMI-only to multiple input features is significant, with the optimal set of input biometrics shown for each condition type (an exhaustive search was conducted to find the input set that maximized AUROC). Comparing BMI-only to multiple input features, we observe clear improvements across different output condition types: AUROC and Prev. Top 25% improve by approximately 10%; model specificity is similar, but model sensitivity improves by 19–26%.

Table 3 illustrates that the tradeoff between specificity and sensitivity can be adjusted by shifting the threshold on the model’s Softmax output up or down. The true positive rate in the row with 25% of examples above the threshold corresponds exactly to the “Prev. Top 25%” column for the multiple-input features, any-condition model in the final row of Table 2 (85.6%).

**Table 3:** Softmax output thresholding to establish binary positive and negative outputs.

Soft-max	Examples		True Pos.		
Threshold	Above Thresh.	Accuracy	Rate	Specificity	Sensitivity
0.00	100.0%	60.5%	60.5%	0.0%	100.0%
0.10	98.6%	61.4%	61.1%	2.9%	99.5%
0.25	82.9%	68.0%	67.2%	31.1%	92.0%
0.31	75.0%	69.3%	69.9%	42.8%	86.6%
0.47	50.0%	67.2%	77.7%	71.8%	64.2%
0.50	46.1%	66.4%	79.3%	75.6%	60.3%
0.66	25.0%	57.7%	86.6%	91.4%	35.7%
0.75	15.2%	51.8%	90.6%	96.4%	22.8%
0.90	2.8%	42.1%	95.8%	99.7%	4.5%
1.00	0.0%	39.5%	100.0%	100.0%	0.0%

The Softmax output of the model, in this case the any-condition ensemble shown in the bottom row of Table 2, can be thresholded to establish binary positive and negative outputs. The training loss function effectively sets the threshold at 0.50 (highlighted row). However, the threshold can be adjusted up or down after training to tradeoff specificity and sensitivity.

**Fig 5:**
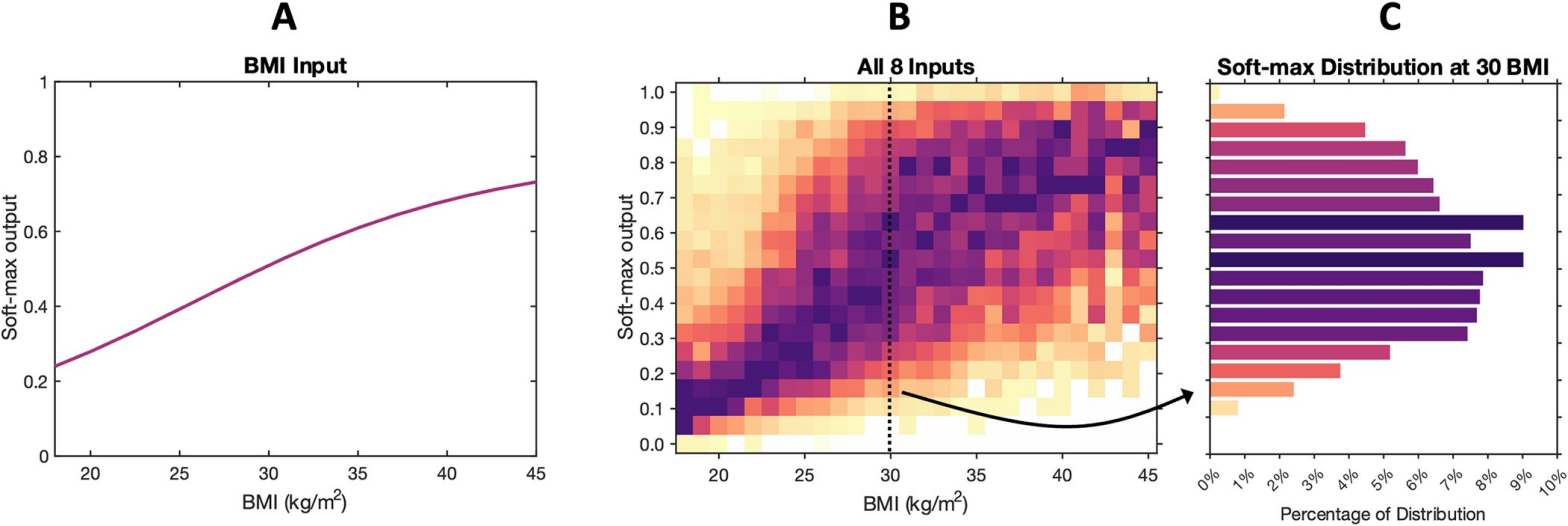
BMI vs. the distribution of Softmax outputs conditioned on BMI for two different models: BMI-only input (A), and all 8 inputs (B). Both models predict the union (any) among nine common health conditions. (C**)** highlights the large stratification of Softmax outputs at 30 BMI for the 8-inputs model.

As shown in Fig 5A, there is a clear relationship between BMI and health risk. However, the BMI model affords only a single risk score (Softmax output) for each BMI input value. BMI alone provides an incomplete picture of health risk. Fig 5B shows a large stratification of health risk (Softmax outputs) from the multiple-biometrics model at each BMI value, especially for 30 BMI, as highlighted in Fig 5C.

Previous approaches to risk estimation tend to stop at the level of predicting the presence/absence of a *specific* condition, a two-class output. We make two advances here: Our machine learning models output condition prevalence, directly, a form of health risk measurement; and our approach is not limited to disease-specific models and can predict the risk of having one or more among a set of conditions.

### Age overwhelms other input features in predicting health risk

**Fig 6:**
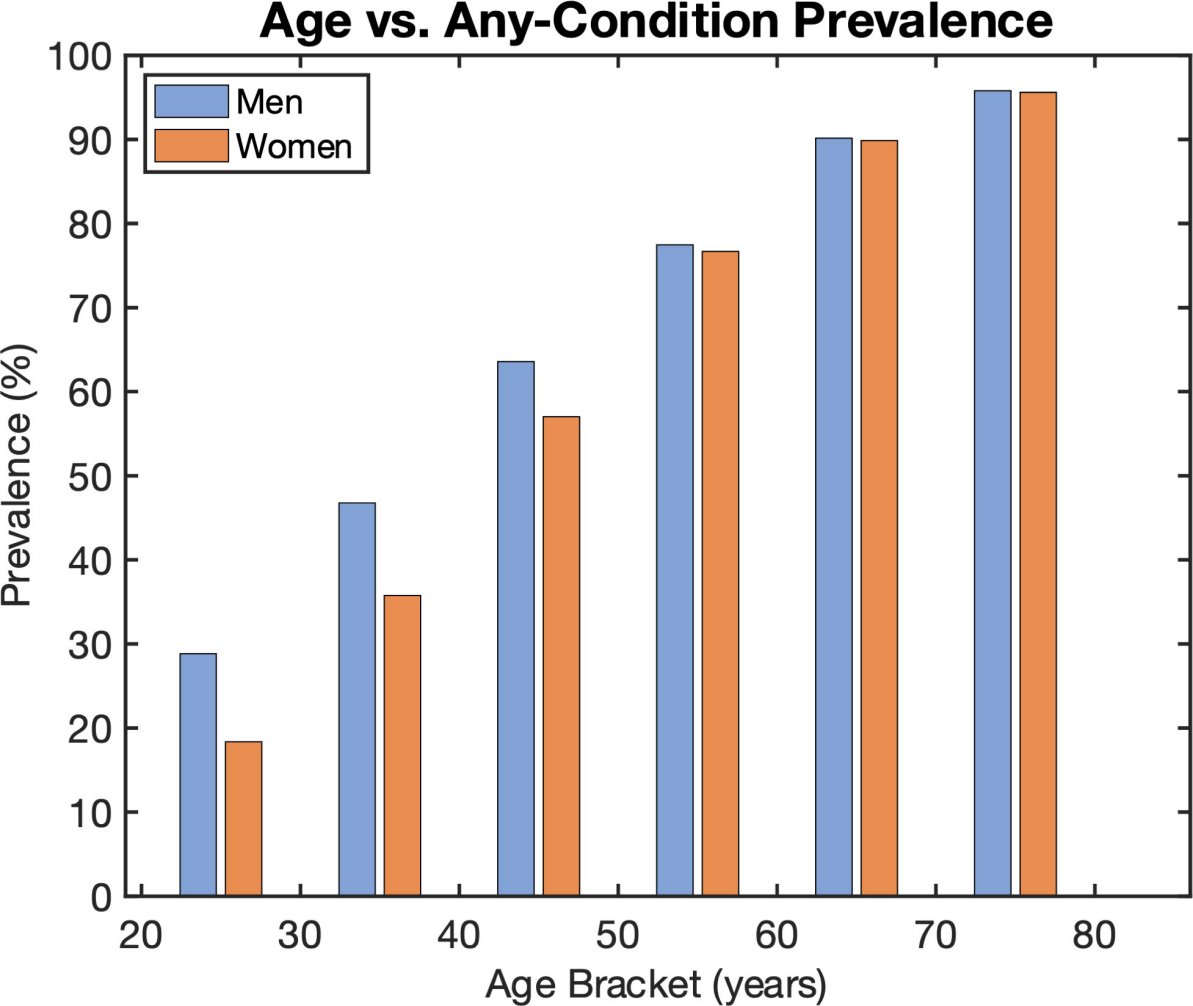
Any-condition prevalence is highly correlated with age.

Age is highly correlated with any-condition health risk, as shown in Fig 6. For example, less than 30% of subjects in the dataset between 20 and 30 years of age have one or more health conditions, whereas 90% or more of subjects age 60 years and above have one or more health condition.

Age overwhelms other variables for health risk prediction (yet cannot be changed with behavior interventions). For example, including age as a model input mutes the differences between {age, BMI} models and {age, multiple biometrics} models. Rather than include age as an input variable, we additionally trained and evaluated age-stratified models, i.e., separate models for ages 18–39, 40–59, and 60+.

In general, we observe that multiple-biometrics models perform significantly better than BMI-only models across age groups, as shown in Table 4. Specificity is generally better for multiple-biometrics models compared to BMI-only models. A notable exception is diabetes prediction among 18- to 39-year-olds: *sensitivity* is similar between BMI-only and multiple-biometrics models, but *specificity* increases significantly for the best set of multiple inputs: waist, thigh, and ethnicity. One possibility is that BMI alone does not distinguish young individuals with higher muscularity from young individuals with higher body fat, whereas waist and thigh circumferences can distinguish the two groups. Among 40–59 and 60+, *specificity* for diabetes prediction is similar, but *sensitivity* increases significantly from BMI-only to multiple-biometrics models. One possibility is that BMI alone fails to identify diabetes risk among many older individuals because it does not indicate how body fat is distributed, whereas waist circumference together with other biometrics can disambiguate visceral fat from subcutaneous fat.

**Table 4:** Comparison of health risk prediction performance using BMI-only input (baseline) and multiple input features for diabetes, hypertension, and condition-agnostic (“Any”) models for different age groups.

Age	Output	Prevalence		Input	AUROC		Prev. Top	Specificity	Sensitivity
Range	Cond. Type	Test	Train	Biomarkers	Test	Train	25% (Test)	(Test)	(Test)
18–39	Diabetes	2.9%	3.2%	BMI	72.7%	68.4%	3.7%	68.3%	63.8%
				Waist, Thigh, Ethnicity	78.7%	76.6%	7.9%	79.7%	65.9%
	Hypertension	25.6%	26.1%	BMI	69.1%	69.2%	20.9%	62.8%	65.7%
				Weight, Sex, Waist, Ethnicity, Height	71.5%	71.7%	46.0%	62.9%	69.5%
	Any	32.8%	32.6%	BMI	64.6%	64.6%	32.4%	63.3%	58.3%
				Waist, Ethnicity, Hip, Sex, Height, Weight, Thigh, PBF	68.2%	70.2%	54.3%	65.1%	61.3%
40–59	Diabetes	12.6%	12.7%	BMI	68.0%	71.0%	16.8%	77.8%	47.3%
				Waist, Ethnicity, Thigh, Height, Sex, Weight, PBF, Hip	77.4%	78.7%	29.8%	76.9%	64.0%
	Hypertension	52.7%	54.2%	BMI	67.5%	64.9%	48.8%	69.5%	55.1%
				Waist, Ethnicity, Thigh, Sex, Height, Weight	67.3%	68.1%	71.8%	65.2%	59.6%
	Any	67.0%	68.1%	BMI	64.0%	63.4%	69.2%	68.6%	52.9%
				Waist, Thigh, Ethnicity, Height, Weight	68.6%	69.1%	85.7%	68.6%	57.8%
60+	Diabetes	24.6%	25.8%	BMI	66.4%	65.4%	31.7%	75.5%	47.5%
				Waist, Ethnicity, Weight, PBF, Height, Sex	72.4%	71.3%	45.5%	74.3%	58.7%
	Hypertension	77.4%	77.6%	BMI	61.8%	61.8%	70.2%	71.3%	45.6%
				PBF, Ethnicity, Waist, Height, Sex, Hip	63.1%	65.2%	86.5%	71.6%	47.9%
	Any	92.3%	93.1%	BMI	61.1%	61.2%	90.8%	62.6%	53.6%
				Waist, Weight, Ethnicity, Thigh, Height, Hip	67.2%	69.4%	97.2%	71.5%	55.5%

For each non-BMI model, we selected the set of multiple input biometrics that produced the highest AUROC test score; inputs are listed in decreasing order of their contribution to AUROC.

### Undiagnosed health conditions are detectable

It is possible to warn people of potential undiagnosed health conditions using a condition-specific model ensemble’s output score. This can be demonstrated for any of the obesity-related diseases included in NHANES, but we limit analysis here to two representative examples—diabetes and hypertension—for the sake of brevity. Our models were trained with target labels that include both diagnosed (11.7% prevalence for diabetes and 34.2% prevalence for hypertension) and undiagnosed (4.4% prevalence for diabetes and 18.7% prevalence for hypertension) conditions.

For the population with diagnosed or undiagnosed diabetes, sensitivity was 68.1%. For the same model, undiagnosed-only diabetes sensitivity was 63.9%, i.e., the majority of undiagnosed diabetes cases were detected by the model. If these models were deployed in the field, these subjects could be alerted to undergo further formal screening to rule out false positives. Similarly, for hypertension the sensitivity in the diagnosed or undiagnosed population was 61.7% and in the undiagnosed population sensitivity was 49.9%.

Including previously undiagnosed conditions also improves model performance. For example, AUROC for diabetes prediction improves from 77.2% to 78.1% after adding previously undiagnosed participants to the positive class during training and testing.

### An equation for health risk from multiple input features

Due to the effectiveness of small neural network models (Result 1), writing them as a set of explicit equations for them becomes practical. This enables convenient implementation and experimentation by non-machine learning practitioners, e.g., using a spreadsheet. See S5 File for details.

### Ensembles are beneficial

**Fig 7:**
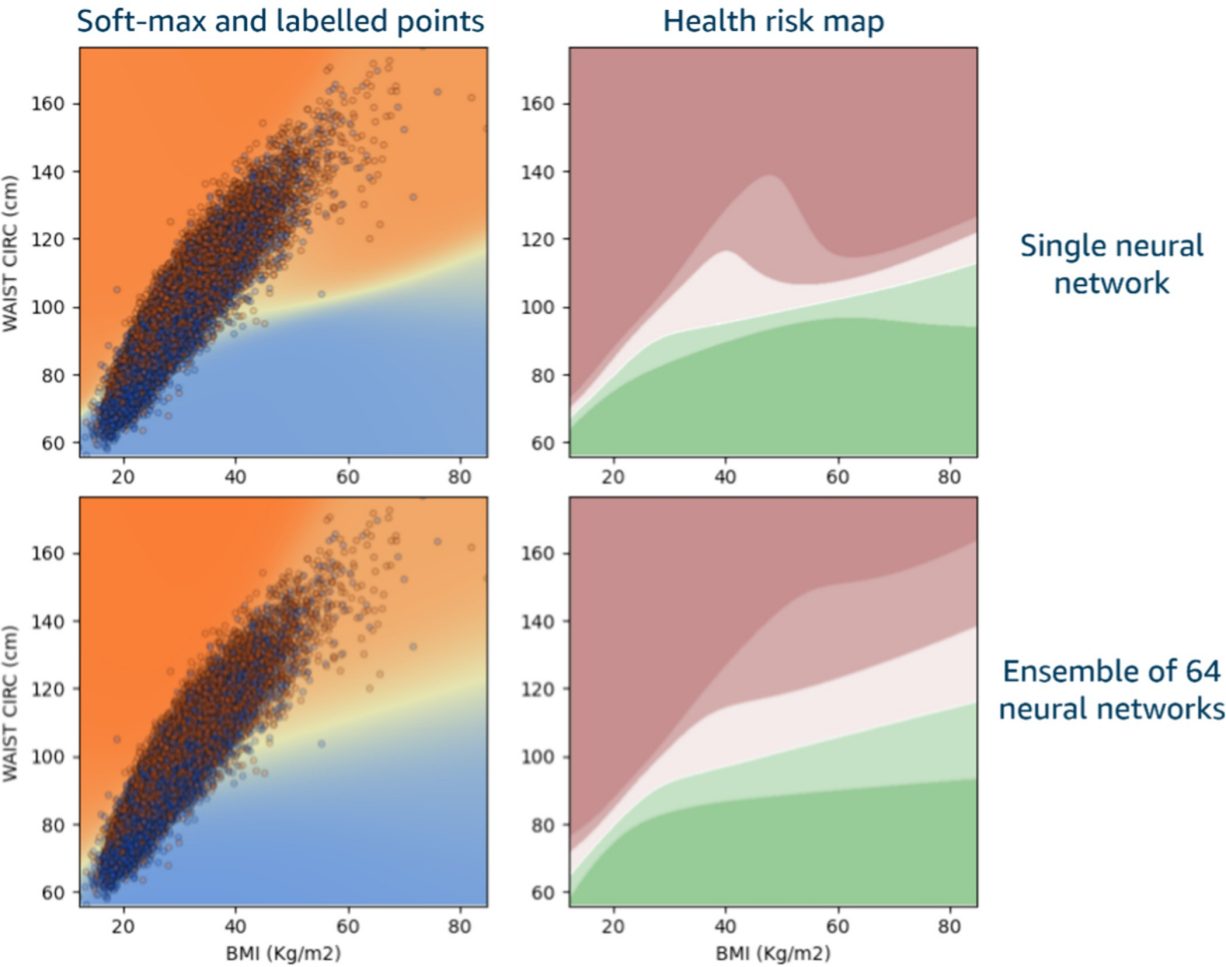
Visualization of improved ensemble generalization compared to single models. The separating surface is similar between single networks and ensembles, but ensembles yield smoother, monotonic risk regions as we move immediately outside the region of high data density.

As illustrated in Fig 7, there is not much difference (in terms of separating surface and risk regions) between single networks and ensembles if we only look within the region where the labeled data lives. However, network ensembles yield much smoother, monotonic risk regions as we move immediately outside the region of high data density. This is an indication that ensembles produce more accurate predictions away from training data, and they can generalize better to previously unseen or slightly unusual data.

**Fig 8:**
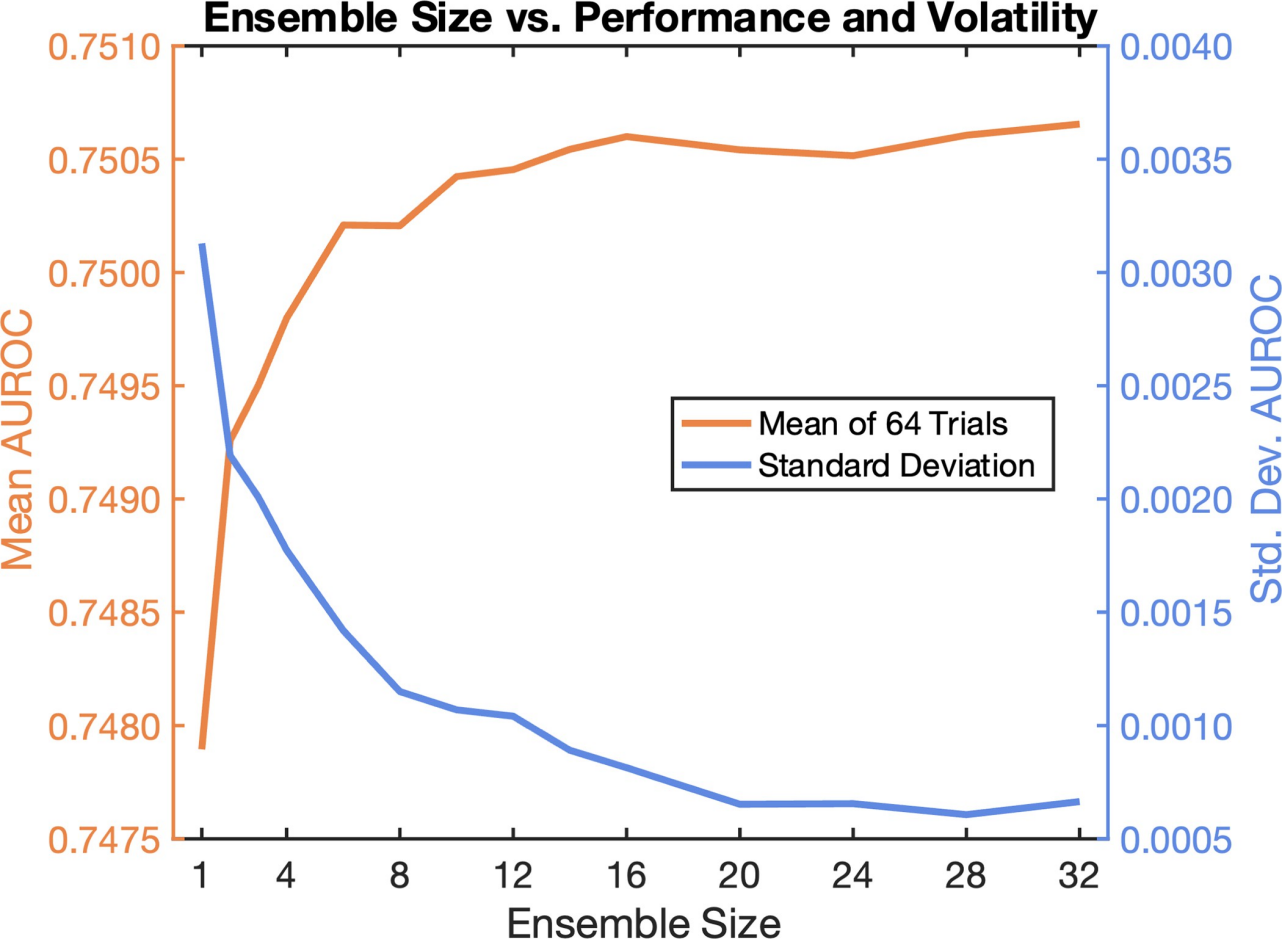
Average performance asymptotically increases (orange) and variability in performance asymptotically decreases (blue) as neural network models are added to the ensemble, as measured across 64 trials for each ensemble size.

As shown in Fig 8, average performance asymptotically improves and variability in performance asymptotically decreases as models are added to the ensemble. All the results in this work were obtained with ensembles of N = 16 neural networks, which balances increased training time and performance (negligible improvements beyond N = 16).

## Limitations

Limitations of the analysis presented here are related to the dataset used (NHANES) and include: examination of cross-sectional data only, no longitudinal studies; establishing statistical associations rather than mechanistic understanding of cause and effect; limited population size; treating diabetes as a single condition without distinction between type I and type II; and use of disease prevalence as a proxy for health risks. Small NNs, although human readable, may still pose challenges for clinical implementation and interpretation; we envision their use primarily in the context of healthcare software applications to manage details in a standardized way. Smartphone-based measurements may introduce different variabilities compared to the traditional measurements from NHANES; large-scale smartphone-based data collection and analysis remain future work.

## Conclusions

We hope that our findings will lead to a better understanding of obesity, its causes, and its effects on people’s health. Our multi-dimensional risk charts and human-readable model equations enable straightforward adoption by medical software developers, clinicians, or health insurers. Finally, combining our findings with emerging technology for body scanning and wearables for tracking activity (e.g., step count) and other behavioral measurements promises to advance the way we assess health risks, track them over time and manage them in a personalized manner.

## Supporting information

S1 Fig Study exclusions diagram. (TIFF)

S1 Table Distribution of the study cohort (after exclusions). (DOCX)

S1 File Health conditions. An explanation of each of the nine common health conditions used in this study.
(DOCX)

S2 File Input features. An explanation of the input features used in this study.
(DOCX)

S3 File Imputation is beneficial. Discussion of imputation results.
(DOCX)

S4 File Data imputation details. (DOCX)

S5 File Explicit equations for neural network. (DOCX)

S6 File Neural network ensembles vs. gradient boosted classification forests. (DOCX)

S7 File Extended tables. Extended versions of Table 2 in the main paper.
(DOCX)
