# Supplementary figures and images for: Modeling health risks using neural network ensembles
Source: PLoS One. 2024 Oct 9;19(10):e0308922. doi: 10.1371/journal.pone.0308922 (PMC11463747; doi:10.1371/journal.pone.0308922)

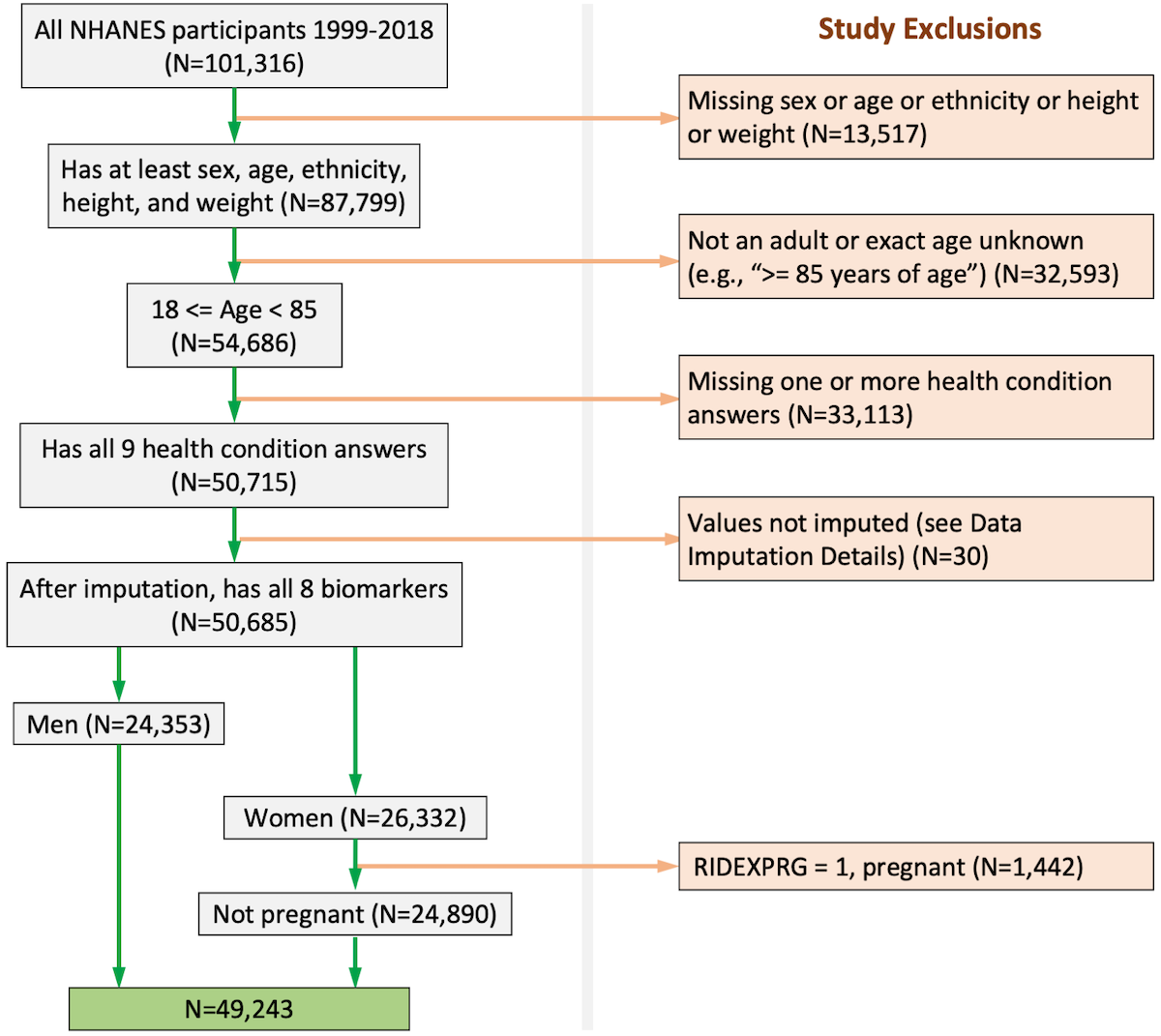

Supplement: S1 Fig — (TIFF) [file pone.0308922.s001.tiff]
