# Supplementary material for: Modeling health risks using neural network ensembles
Source: PLoS One. 2024 Oct 9;19(10):e0308922. doi: 10.1371/journal.pone.0308922 (PMC11463747; doi:10.1371/journal.pone.0308922)
Supplement: S1 File — An explanation of each of the nine common health conditions used in this study. (DOCX) [file pone.0308922.s003.docx]

**Health conditions**

All analyses in this study were conducted using nine common health conditions from the National Health and Nutrition Survey (NHANES) dataset, described below.

- **Hypertension:** a “yes” answer to the question “Have you ever been told by a doctor or other health professional that you had hypertension, also called high blood pressure?” (“BPQ020” in NHANES) or systolic blood pressure ≥130 or diastolic blood pressure ≥80.
- **Diabetes:** a “yes” answer to the question “Other than during pregnancy, have you ever been told by a doctor or health professional that you have diabetes or sugar diabetes?” (“DIQ010” in NHANES) or fasting glucose ≥7.0 mmol/L or glycohemoglobin ≥6.5% or glucose tolerance test ≥11.1 mmol/L.
- **Arthritis:** a “yes” answer to the question “Has a doctor or other health professional ever told you that you had arthritis?” (“MCQ160a” in NHANES).
- **Coronary Heart Disease:** a “yes” answer to the question “Has a doctor or other health professional ever told you that you had coronary heart disease?” (“MCQ160c” in NHANES).
- **Angina:** a “yes” answer to the question “Has a doctor or other health professional ever told you that you had angina, also called angina pectoris?” (“MCQ160d” in NHANES).
- **Congestive Heart Failure:** a “yes” answer to the question “Has a doctor or other health professional ever told you that you had congestive heart failure?” (“MCQ160b” in NHANES).
- **Had a Heart Attack:** a “yes” answer to the question “Has a doctor or other health professional ever told you that you had a heart attack (also called myocardial infarction)?” (“MCQ160e” in NHANES).
- **Had a Stroke:** a “yes” answer to the question “Has a doctor or other health professional ever told you that you had a stroke?” (“MCQ160f” in NHANES).
- **Cancer (General Malignancy):** a “yes” answer to the question “Have you ever been told by a doctor or other health professional that you had cancer or a malignancy of any kind?” (“MCQ220” in NHANES).
