# Supplementary material for: Modeling health risks using neural network ensembles
Source: PLoS One. 2024 Oct 9;19(10):e0308922. doi: 10.1371/journal.pone.0308922 (PMC11463747; doi:10.1371/journal.pone.0308922)
Supplement: S2 File — An explanation of the input features used in this study. (DOCX) [file pone.0308922.s004.docx]

**Biomarkers (input features)**

All analyses in this study were conducted using biomarkers from the National Health and Nutrition Survey (NHANES) dataset (https://wwwn.cdc.gov/nchs/nhanes/).

- **Body Mass Index (BMI):** calculated as weight in kilograms divided by height in meters squared (“BMXBMI” in NHANES).
- **Percentage Body Fat (PBF):** percentage of body mass that comes from fat as measured by Dual-energy X-ray absorptiometry (DEXA) (“DXDTOPF” in NHANES).
- **Waist Circumference:** circumference in centimeters of the uppermost lateral border of the ilium using a tape measure (“BMXWAIST” in NHANES).
- **Thigh Circumference:** circumference in centimeters around the midpoint of the thigh (“BMXTHICR” in NHANES).
- **Hip Circumference:** circumference along a horizontal plane positioned at the maximum protuberance of the buttocks when viewed in profile (“BMXHIP” in NHANES).
- **Height:** standing body height in centimeters (“BMXHT” in NHANES).
- **Weight:** body weight in kilograms measured using a digital weight scale (“BMXWT” in NHANES).
- **Age:** age in years (“RIDAGEYR” in NHANES),
- **Sex:** male or female (“RIAGENDR” in NHANES).
- **Ethnicity:** “Mexican American”, “Other Hispanic”, “Non-Hispanic White”, “Non-Hispanic Black”, or “Other Race – Including Multi-Racial” (“RIDRETH1” in NHANES).
