# Supplementary material for: Modeling health risks using neural network ensembles
Source: PLoS One. 2024 Oct 9;19(10):e0308922. doi: 10.1371/journal.pone.0308922 (PMC11463747; doi:10.1371/journal.pone.0308922)
Supplement: S3 File — Discussion of imputation results. (DOCX) [file pone.0308922.s005.docx]

**Imputation is beneficial**

Imputing missing input values in NHANES during training yields better health risk prediction models. For example, NHANES includes only 2223 examples with valid values for sex, ethnicity, height, weight, percent body fat, waist circumference, and hip circumference. Training and testing a conditioning agnostic model using these examples (with 40% train, 20% validation, and 40% test partitions) yields a train set AUROC of 76.2% and a significantly lower test set AUROC of 70.8%, indicating some model overfitting. Including imputations, mainly for hip, provides 49243 examples. Training and testing a condition agnostic model using these examples (with 40% train, 20% validation, and 40% test partitions) yields a train set AUROC of 74.5%, which is lower than the no-imputations model (imputations are imperfect), but yields a test set AUROC of 73.6%, which is significantly higher than the no-imputations model, indicating better generalization. Test set prevalence among the top 25% Softmax outputs, precision, and recall are also significantly higher for the model trained with imputations. This is summarized in **Table A** below.

**Table A.** Imputing missing input values in NHANES during training yields better health risk prediction models.
