# Supplementary material for: Modeling health risks using neural network ensembles
Source: PLoS One. 2024 Oct 9;19(10):e0308922. doi: 10.1371/journal.pone.0308922 (PMC11463747; doi:10.1371/journal.pone.0308922)
Supplement: S4 File — (DOCX) [file pone.0308922.s006.docx]

**Data imputation details**

**Architecture**

To impute missing values in NHANES, we used an ensemble of 20 hourglass-shaped autoencoder neural networks. Each autoencoder included five layers. Each layer consisted of a linear (a.k.a. fully-connected) layer followed by a “Swish” activation layer [1] and a batch normalization layer [2]. The layer widths (number of nodes) were: 224 🡪 112 🡪 56 🡪 112 🡪 224. This architecture was chosen empirically, i.e., multiple network depths and widths were tested, and the best-performing network architecture was selected. We used 112 biometric and demographic dimensions from NHANES as input/output, which are listed at the end of this section. Each input was paired with a binary (0/1) flag indicating if the value was available in NHANES for a specific subject (1) or not (0), yielding 224 total input and output dimensions.

**Training**

The Adam optimization algorithm was used to train each network. We imposed an L1 loss on every value output by the network that had an available ground truth from NHANES. To teach the network to fill in (impute) missing values for each example (subject), we randomly set input flags to zero and replaced input values with their global average. When randomly omitting inputs, we found it important to capture actual correlations in the dataset with respect to missing values, and so we collected all valid input masks from the training dataset as exemplars to apply during training. Input masks were defined as the intersection of the actual example valid input mask and an exemplar valid input mask randomly selected from the training dataset, i.e., the number of available input values was strictly less than or equal to the number of available input values. All zero-flag inputs were replaced with their global average.

After training an ensemble of networks in this fashion, we trained a new ensemble, but instead of replacing missing inputs with their global average, we replaced them with the previous ensemble’s imputed value. This iterative approach was repeated several times until convergence.

Imputed values used in the main paper were generated via five-fold cross validation. Only training examples from the main paper were used to train and validate imputation ensembles. Training examples were divided into five disjoint equal-size groups. For each validation group, we used the other 4/5ths of the dataset to train 4 neural networks. This yielded a total ensemble of 4 x 5 = 20 neural networks that could be used to impute values in the validation and test sets used in the main paper.

**Imputation Inputs/Outputs**

1. BMXWT Weight (Kg)
2. BMXHT Height (cm)
3. BMXTHICR Thigh Circumference (cm)
4. BMXWAIST Waist Circumference (cm)
5. BMXHIP Hip Circumference (cm)
6. BMXARMC Arm Circumference (cm)
7. BMXLEG Upper leg length (cm)
8. BMXCALF Maximum calc circumference (cm)
9. BMXTRI Triceps skinfold (mm)
10. BMXSUB Subscapular Skinfold (mm)
11. BMXRECUM Recumbent Length (cm)
12. BMXSAD1 Sagittal Abdominal Diameter 1st (cm)
13. BMXSAD2 Sagittal Abdominal Diameter 2nd (cm)
14. BMXSAD3 Sagittal Abdominal Diameter 3rd (cm)
15. BMXSAD4 Sagittal Abdominal Diameter 4th (cm)
16. BMDAVSAD Average Sagittal Abdominal Diameter (cm)
17. BMXBMI BMI (Kg/m2)
18. DXDTOPF Percent Total Body Fat (%)
19. DXDTOFAT Body Fat Mass (g)
20. DXDTRPF Percent Trunk Fat (%)
21. DXXRLFAT Right Leg Fat (g)
22. DXXRAFAT Right Arm Fat (g)
23. DXXLLFAT Left Leg Fat (g)
24. DXXLAFAT Left Arm Fat (g)
25. DXDLAPF Percent Fat Left Arm (%)
26. DXDRAPF Percent Fat Right Arm (%)
27. DXDLLPF Percent Fat Left Leg (%)
28. DXDRLPF Percent Fat Right Leg (%)
29. DXDTOBMC Total Body Mineral Content (g)
30. DXDTOBMD Total Body Mineral Density (g/cm^2)
31. DXXHEA Head Area (cm^2)
32. DXXHEBMC Head Bone Mineral Content (g)
33. DXXHEBMD Head Bone Mineral Density (g/cm^2)
34. DXXHEFAT Head Fat (g)
35. DXDHELE Head Lean excl BMC (g)
36. DXXHELI Head Lean incl BMC (g)
37. DXDHETOT Head Total (g)
38. DXDHEPF Head Percent Fat
39. DXXLAA Left Arm Area (cm^2)
40. DXXLABMC Left Arm BMC (g)
41. DXXLABMD Left Arm BMD (g/cm^2)
42. DXDLALE Left Arm Lean excl BMC (g)
43. DXXLALI Left Arm Lean incl BMC (g)
44. DXDLATOT Left Arm Total (g)
45. DXXLLA Left Leg Area (cm^2)
46. DXXLLBMC Left Leg BMC (g)
47. DXXLLBMD Left Leg BMD (g/cm^2)
48. DXDLLLE Left Leg Lean excl BMC (g)
49. DXXLLLI Left Leg Lean incl BMC (g)
50. DXDLLTOT Left Leg Total (g)
51. DXXRAA Right Arm Area (cm^2)
52. DXXRABMC Right Arm BMC (g)
53. DXXRABMD Right Arm BMD (g/cm^2)
54. DXDRALE Right Arm Lean excl BMC (g)
55. DXXRALI Right Arm Lean incl BMC (g)
56. DXDRATOT Right Arm Total (g)
57. DXXRLA Right Leg Area (cm^2)
58. DXXRLBMC Right Leg BMC (g)
59. DXXRLBMD Right Leg BMD(g/cm^2)
60. DXDRLLE Right Leg Lean excl BMC (g)
61. DXXRLLI Right Leg Lean incl BMC (g)
62. DXDRLTOT Right Leg Total (g)
63. DXXLRA Left Ribs Area (cm^2)
64. DXXLRBMC Left Ribs BMC (g)
65. DXXLRBMD Left Ribs BMD (g/cm^2)
66. DXXRRA Right Ribs Area (cm^2)
67. DXXRRBMC Right Ribs BMC (g)
68. DXXRRBMD Right Ribs BMD (g/cm^2)
69. DXXTSA Thoracic Spine Area (cm^2)
70. DXXTSBMC Thoracic Spine BMC (g)
71. DXXTSBMD Thoracic Spine BMD (g/cm^2)
72. DXXLSA Lumbar Spine Area (cm^2)
73. DXXLSBMC Lumbar Spine BMC (g)
74. DXXLSBMD Lumbar Spine BMD (g/cm^2)
75. DXXPEA Pelvis Area (cm^2)
76. DXXPEBMC Pelvis BMC (g)
77. DXXPEBMD Pelvis BMD (g/cm^2)
78. DXDTRA Trunk Bone area (cm^2)
79. DXDTRBMC Trunk BMC (g)
80. DXDTRBMD Trunk Bone BMD (g/cm^2)
81. DXXTRFAT Trunk Fat (g)
82. DXDTRLE Trunk Lean excl BMC (g)
83. DXXTRLI Trunk Lean incl BMC (g)
84. DXDTRTOT Trunk Total (g)
85. DXDSTA Subtotal Area (cm^2)
86. DXDSTBMC Subtotal BMC (g)
87. DXDSTBMD Subtotal BMD (g/cm^2)
88. DXDSTFAT Subtotal Fat (g)
89. DXDSTLE Subtotal Lean excl BMC (g)
90. DXDSTLI Subtotal Lean incl BMC (g)
91. DXDSTTOT Subtotal (Total excl Head) (g)
92. DXDSTPF Subtotal Percent Fat
93. DXDTOA Total Area (cm^2)
94. DXDTOLE Total Lean excl BMC (g)
95. DXDTOLI Total Lean incl BMC (g)
96. DXDTOTOT Total Lean+Fat (g)
97. DXXANFM Android fat mass
98. DXXANLM Android lean mass
99. DXXANTOM Android total mass
100. DXXGYFM Gynoid fat mass
101. DXXGYLM Gynoid lean mass
102. DXXGYTOM Gynoid total mass
103. DXXAGRAT Android to Gynoid ratio
104. DXXAPFAT Android percent fat
105. DXXGPFAT Gynoid percent fat
106. RIDAGEYR Age (years)
107. RIDRETH1 Ethnicity
108. SMD057 Cigarettes smoked per day when quit
109. SMQ020 Smoked at least 100 cigs in life
110. SMQ040 Now smokes
111. SMD650 Average number of cigarettes smoked in past 30 days
112. RIAGENDR Sex (female, male)

**References**

1. Ramachandran P, Zoph B, Le QV. Searching for activation functions. arXiv:1710.05941 2017

2. Ioffe S, Szegedy C. Batch normalization: accelerating deep network training by reducing internal covariate shift. Proc. of the 32^nd^ International Conference on Machine Learning. 2015 Jul 7-9. Lille, France.
