# Supplementary material for: Modeling health risks using neural network ensembles
Source: PLoS One. 2024 Oct 9;19(10):e0308922. doi: 10.1371/journal.pone.0308922 (PMC11463747; doi:10.1371/journal.pone.0308922)
Supplement: S5 File — (DOCX) [file pone.0308922.s007.docx]

**Explicit equations for neural network**

In this section, we step through one of the neural networks in the eight-input, any-condition ensemble described in the main paper in the **Methods: Neural networks** and **Results and Discussion: Small neural networks are effective for health risk prediction** sections.

It is possible to write a single equation that represents the network. However, for greater clarity, we break the overall computation into steps, corresponding to the sequential computation stages of the neural network. For each stage of the network, we write an explicit, human-readable equation that includes all the parameters (coefficients/weights and biases) learned automatically during model training. These equations, which faithfully describe the internal mathematical operations of the neural network, allow it to be implemented outside of a machine learning framework, e.g., in a spreadsheet. The network uses five basic mathematical operations: add, subtract, multiply, divide, and the exponential function (exp).

The output of each stage is used as the input to the next stage. For example, in the hidden layer step, the outputs are called $a$ and $b$ and are shown on the righthand side of each equation’s equal (=) sign; $a$ is the input to the first equation in the subsequent “activation function” step, which produces an output called $c$, which is shown on the lefthand side of the equation’s equal (=) sign, and so on.

**Step 1: Assemble the inputs**

$\mathrm{waist}$ is waist circumference in centimeters

$\mathrm{thigh}$ is thigh circumference in centimeters

$\mathrm{ethnicity}_{MA}$ is 1 if race/Hispanic origin is Mexican American and 0 otherwise

$\mathrm{ethnicity}_{OH}$ is 1 if race/Hispanic origin is other Hispanic and 0 otherwise

$\mathrm{ethnicity}_{NHW}$ is 1 if race/Hispanic origin is non-Hispanic white and 0 otherwise

$\mathrm{ethnicity}_{NHB}$ is 1 if race/Hispanic origin is non-Hispanic black and 0 otherwise $\mathrm{ethnicity}_{OR}$ is 1 if other race including multi-racial and 0 otherwise

$\mathrm{height}$ is body height in cm

$\mathrm{sex}$ is 1 if female and 0 if male

$\mathrm{weight}$ is body weight in kg

$\mathrm{pbf}$ is percentage body fat

$\mathrm{hip}$ is hip circumference in centimeters

Ethnicity is encoded as a five-valued variable in NHANES, e.g., RIDRETH1=1 means “Mexican American,” RIDRETH1=2 means “Other Hispanic,” etc. This type of categorical variable is not ideal for a neural network, i.e., the values are categorical (order does not imply direction), but the network expects scalar inputs. Therefore, in practice, ethnicity is separated into five indicator values, e.g., $\mathrm{ethnicity}_{MA}$ is 1 (with all other ethnicity indicator values set to 0) if the subject is Mexican American.

The values assembled in this step should be substituted for the corresponding variable name in the next step.

**Step 2: Input normalization**

In this step we normalize each input values so that, on average, each will have zero mean and unit variance. Specifically, for each continuous input variable, we subtract the overall mean and divide by the overall standard deviation. The mean and standard deviation were computed on the training set during the model training procedure. Mean and standard deviation values are provided in each equation below. Inputs are shown on the righthand side of each equal sign (=), and outputs are shown on the lefthand side of each equal sign with an added circumflex symbol above the output variable name to further disambiguate it from the input. For example, “$\mathrm{waist}$” is the input to the first equation, and “$\hat{\mathrm{waist}}$” is the output. As in the previous step, each output from this step will be used as an input to the following step.

$$\hat{\mathrm{waist}}=\left( waist-98.99 \right)\div16.06$$

$$\hat{\mathrm{thigh}}=\left( thigh-54.78 \right)\div6.28$$

$\hat{\mathrm{ethnicity}_{MA}}=(\mathrm{ethnicity}_{MA}-0.5)\div0.5$

$\hat{\mathrm{ethnicity}_{OH}}=(\mathrm{ethnicity}_{OH}-0.5)\div0.5$

$\hat{\mathrm{ethnicity}_{NHW}}=(\mathrm{ethnicity}_{NHW}-0.5) \div0.5$

$\hat{\mathrm{ethnicity}_{NHB}}=(\mathrm{ethnicity}_{NHB}-0.5) \div0.5$

$\hat{\mathrm{ethnicity}_{OR}}={(ethnicity}_{OR}-0.5) \div0.5$

$$\hat{\mathrm{height}}=\left( height-167.13 \right)\div10.22$$

$\hat{\mathrm{sex}}=(sex-0.5)\div0.5$

$$\hat{\mathrm{weight}}=\left( weight-81.25 \right)\div21.10$$

$$\hat{\mathrm{pbf}}=\left( pbf-34.99 \right)\div7.53$$

$$\hat{\mathrm{hip}}=\left( hip-108.78 \right)\div9.50$$

**Step 3: Hidden layer**

This operation can be explained as a pair of linear regressors (two nodes), each with the form $y=\mathbf{m}^{T}\mathbf{x}+\mathrm{bias}$, where $\mathbf{x}$ is a vector representation of the inputs to this step (outputs from the previous step), $\mathbf{m}$ is a vector of coefficients (weights), $\mathrm{bias}$ is a bias (offset) value, and $y$ is the output. The $\mathbf{m}^{T}\mathbf{x}$ operation is the dot product (multiply and then sum) between the model weights and the input values, i.e., $\mathbf{m}^{T}\mathbf{x=}m_{\mathrm{waist}}\hat{\mathrm{waist}}+m_{\mathrm{thigh}}\hat{\mathrm{thigh}}+\ldots+m_{\mathrm{weight}}\hat{\mathrm{weight}}$. $\mathbf{m}$ and $b$ are automatically learned during model training. The two equations below are written in long form, with all multiplications and additions shown explicitly. Terms with coefficients/weights with a magnitude less than 0.00001 are omitted for simplicity.

$$a= 0.1935\cdot\hat{\mathrm{waist}}+0.2642\cdot\hat{\mathrm{thigh}}+0.0040\hat{{\cdot ethnicity}_{NHW}}+0.0337\hat{{\cdot ethnicity}_{NHB}}-0.0059\cdot\hat{\mathrm{ethnicity}_{OR}}-0.0428\cdot\hat{\mathrm{sex}}-0.1741\cdot\hat{\mathrm{weight}}+0.1006\cdot\hat{\mathrm{pbf}}-0.0691\cdot\hat{\mathrm{hip}}+3.7168$$

$$b= 0.0547\cdot\hat{\mathrm{waist}}-0.3442\cdot\hat{\mathrm{thigh}}-0.0624\hat{{\cdot ethnicity}_{MA}}-0.0270\hat{{\cdot ethnicity}_{OH}}+0.0224\cdot\hat{\mathrm{ethnicity}_{NHW}}+0.0298\hat{{\cdot ethnicity}_{NHB}}-0.0489\cdot\hat{\mathrm{height}}-0.0060\cdot\hat{\mathrm{sex}}+0.1069\cdot\hat{\mathrm{weight}}-0.0600\cdot\hat{\mathrm{pbf}}+0.0993\cdot\hat{\mathrm{hip}}-3.0516$$

**Step 4: Activation function (sigmoid)**

This step adds nonlinearity to the model. The two inputs to this step are $a$ and $b$, which are the outputs from the previous step, and the outputs of this step are called $c$ and $d.$

$$c= \frac{1}{1+exp(-a)}$$

$$d= \frac{1}{1+exp(-b)}$$

**Step 5: Batch normalization**

This step helps the model train faster, and more stably. In a nutshell, the values in the batch normalization are found automatically during training, and they standardize the inputs to the following step, i.e., so that they are not too large or too small relative to one another. The inputs to this step are $c$ and $d$, which are the outputs from the previous step. The outputs of this step are on the lefthand side of each equal (=) sign, and are denoted with an additional circumflex symbol (^) to disambiguate them from the inputs.

$$\begin{matrix} \hat{c} & = & \frac{c-0.9750}{0.0073}\cdot5.2292-0.2822 \\ & = & 711.7162\cdot c-694.2056 \end{matrix}$$

$$\begin{matrix} \hat{d} & = & \frac{d-0.0480}{0.0104}\cdot3.8676-0.2432 \\ & = & 370.6302\cdot d-18.0358 \end{matrix}$$

**Step 6: Output layer**

Similar to step 3, this step works like a linear regressor, mapping the outputs from the previous stage to the inputs of the next stage via a linear equation. This step involves two linear regressors (nodes). Each equation shares the same two inputs, $\hat{c}$ and $\hat{d}$, but uses a different set of learned coefficients/weights and bias to produce a different output value. The two outputs are called $\mathrm{score}_{\mathrm{neg}}$, which is the negative class score (i.e., negative to the condition), and $\mathrm{score}_{\mathrm{pos}}$, which is the positive class score (i.e., positive to the condition).

$$\mathrm{score}_{\mathrm{neg}}= -0.285016\cdot\hat{c}-0.371998\cdot\hat{d}-0.3820$$

$$\mathrm{score}_{\mathrm{pos}}= 0.000069\cdot\hat{c}+0.010498\cdot\hat{d}-0.2156$$

**Step 7: Softmax output**

The final step is to convert the $\mathrm{score}_{\mathrm{neg}}$ and $\mathrm{score}_{\mathrm{pos}}$ outputs from the previous step into probability values, i.e., positive and sum to one. Since we care most about the probability of being *positive* to a condition, we omit the $\mathrm{softmax}_{\mathrm{neg}}$ equation for simplicity. Note that $\mathrm{softmax}_{\mathrm{neg}}$ can be computed simply as $\mathrm{softmax}_{\mathrm{neg}}=1-\mathrm{softmax}_{\mathrm{pos}}.$

$$\mathrm{softmax}_{\mathrm{pos}}= \frac{exp(\mathrm{score}_{\mathrm{pos}})}{\exp\left( \mathrm{score}_{\mathrm{pos}} \right)+exp(\mathrm{score}_{\mathrm{neg}})}$$

During training, the binary cross entropy loss is defined over $\mathrm{softmax}_{\mathrm{pos}}$ and $\mathrm{softmax}_{\mathrm{neg}}$:

$$\mathrm{loss}=-\sum_{i}^{N} w_{i}\left( y_{i} log(\mathrm{softmax}_{i,pos}) + (1-y_{i}) log(\mathrm{softmax}_{i,neg}) \right)$$

where $i$ is the training example index, $N$ is the number of training examples, $y_{i}$ is the ground truth condition label (0 for negative to condition, and 1 for positive to condition), and $w_{i}$ is the sample weight. During inference, $\mathrm{softmax}_{\mathrm{pos}}$ can be used as a health risk score.

**Step 8: Thresholding (optional)**

If a “hard” classification is desired, $\mathrm{softmax}_{\mathrm{pos}}$ can be thresholded to obtain a positive or negative output, i.e., less than the threshold is negative to the condition and greater than the threshold is positive to the condition. The training loss effectively sets the threshold at 0.5. However, it can be shifted up or down after training to tradeoff specificity and sensitivity as shown in **Table 3** in the main paper.

**Example**

Inputs:

$\mathrm{waist}=98.3 \mathrm{cm}$

$thigh=52.9\mathrm{cm}$

$\mathrm{ethnicity}_{MA}=1$ (Mexican American)

$\mathrm{ethnicity}_{OH}=0$

$\mathrm{ethnicity}_{NHW}=0$

$\mathrm{ethnicity}_{NHB}=0$

$\mathrm{ethnicity}_{OR}=0$

$height=162 cm$

$sex=1$ (female)

$weight=77 kg$

$\mathrm{pbf}=32.2\%$ (percent body fat)

$hip=88.5 cm$

Input normalization:

$\hat{\mathrm{waist}}=\left( waist-98.99 \right)\div16.06=-0.042964$

$\hat{\mathrm{thigh}}=\left( thigh-54.78 \right)\div6.28=-0.299363$

$\hat{\mathrm{ethnicity}_{MA}}=\left( \mathrm{ethnicity}_{MA}-0.5 \right)\div0.5=1$

$\hat{\mathrm{ethnicity}_{OH}}=\left( \mathrm{ethnicity}_{OH}-0.5 \right)\div0.5=-1$

$\hat{\mathrm{ethnicity}_{NHW}}=\left( \mathrm{ethnicity}_{NHW}-0.5 \right)\div0.5=-1$

$\hat{\mathrm{ethnicity}_{NHB}}=\left( \mathrm{ethnicity}_{NHB}-0.5 \right)\div0.5=-1$

$\hat{\mathrm{ethnicity}_{OR}}={(ethnicity}_{OR}-0.5) \div0.5=-1$

$\hat{\mathrm{height}}=\left( height-167.13 \right)\div10.22=-0.501957$

$\hat{\mathrm{sex}}=\left( sex-0.5 \right)\div0.5=1$

$\hat{\mathrm{weight}}=\left( weight-81.25 \right)\div21.10=-0.201422$

$\hat{\mathrm{pbf}}=\left( pbf-34.99 \right)\div7.53=-0.370518$

$\hat{\mathrm{hip}}=\left( hip-108.78 \right)\div9.50=-2.134737$

Hidden layer:

$a= 3.704099$

$b= -3.231244$

Activation function (sigmoid):

$c= 0.975969$

$d= 0.038007$

Batch normalization:

$\hat{c}=0.407348$

$\hat{d}=-3.949258$

Output layer:

$\mathrm{score}_{\mathrm{neg}}= 0.971015$

$\mathrm{score}_{\mathrm{pos}}= -0.257031$

Softmax:

$\mathrm{softmax}_{\mathrm{pos}}= 0.226524$ (zero-to-one scale)

Thresholding (optional):

Because $\mathrm{softmax}_{\mathrm{pos}}<0.5$ the predicted classification is “negative to conditions”

**Implementing these equations in a spreadsheet**

As an example, here we show how to implement **Step 3: Hidden layer** in a spreadsheet. Each of the other steps can similarly be implemented in the same spreadsheet. The outputs from each step are stored in a set of cell assigned to that step, and the output values are computed via spreadsheet equations (e.g., “=SUM(A2:A8)”, “=(A9-A10)/A11”). Subsequent steps then use the values in these cells as their inputs. In this way, equations (steps) can be chained together as outlined above. A screenshot of the spreadsheet is shown in **Table A** below.

**Table A. Screenshot of a spreadsheet that implements the equations (steps) in this file.**
