# Supplementary material for: Modeling health risks using neural network ensembles
Source: PLoS One. 2024 Oct 9;19(10):e0308922. doi: 10.1371/journal.pone.0308922 (PMC11463747; doi:10.1371/journal.pone.0308922)
Supplement: S6 File — (DOCX) [file pone.0308922.s008.docx]

**Neural Network Ensembles vs. Gradient Boosted Classification Forests**

**Fig A** in this supplementary file provides a comparison of model performance, as measured by AUROC, between the neural network ensembles proposed in this work, and gradient boosted classification forests [1], which is a popular machine learning approach for handling data similar to NHANES. For example, see Zhou et al. [2], Dinh et al. [3] and MacNell et al. [4]. We used the open-source XGBoost Python library (<https://github.com/dmlc/xgboost>) to generate comparison results.

In all cases, models were trained with eight inputs and any-condition outputs, as described in the main paper. Each neural network includes a single two-node hidden layer. The neural network ensembles trend line (orange) corresponds to ensemble sizes of N=1 to N=16 models. Each classification forest has two key hyperparameters: (1) maximum depth, and (2) number of trees. We varied both of these hyperparameters and recorded the AUROC on the test partition after training each classification forest**.** Each XGBoost uniform-colored trend line (blues and greens) corresponds to the same maximum depth, and each trend is over the number of trees, which was varied from N=1 to N=50 trees.

We see that neural network ensembles attain higher AUROC on this dataset and task, especially at lower model parameter budgets. For example, with a 40-parameter budget, a single neural network (N=1) achieves an AUROC of 0.747 compared to 0.711 for a boosted classifier forest with maximum depth of 2 and N=3 trees. We see that boosted classification forests overfit beyond approximately 700 parameters for this dataset and task.


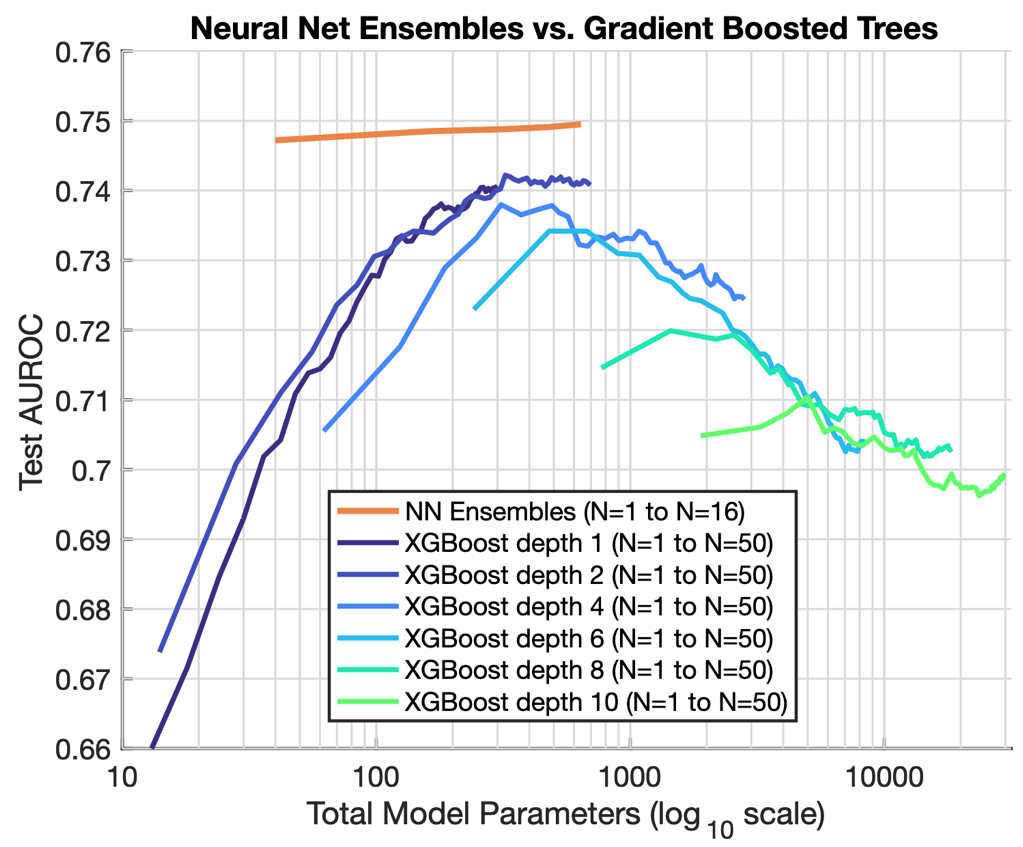


**Fig A. Neural Network Ensembles vs. Gradient Boosted Classification Forests**. Comparison of model performance, as measured by AUROC, between the neural network ensembles proposed in this work, and gradient boosted classification forests, which is a popular machine learning approach for handling data similar to NHANES.

**References**

1. Friedman JH. Greedy function approximation: A gradient boosting machine. Ann. Statist. 2001;29:1189-1232. doi: 10.1214/aos/1013203451

2. Zhou W, Eckler S, Barszczyk A, Waese-Perlman A, Wang Y, Gu X, et al. Waist circumference prediction for epidemiological research using gradient boosted trees. BMC Medical Research Methodology. 2021;21:47. doi: 10.1186/s12874-021-01242-9.

3. Dinh A, Miertschin S, Young A, Mohanty SD. A data-driven approach to predicting diabetes and cardiovascular disease with machine learning. BMC Medical Informatics and Decision Making. 2019;19:211. doi: [10.1186/s12911-019-0918-5](https://doi.org/10.1186/s12911-019-0918-5).

4. MacNell N, Feinstein L, Wilkerson J, Salo PM, Mosberry SA, Fessler MB, *et al.* Implementing machine learning methods with complex survey data: lessons learned on the impacts of accounting sampling weights in gradient boosting. PLoS ONE. 2023;18:e0280387. doi: 10.1371/journal.pone.0280387.
