# Supplementary material for: Modeling health risks using neural network ensembles
Source: PLoS One. 2024 Oct 9;19(10):e0308922. doi: 10.1371/journal.pone.0308922 (PMC11463747; doi:10.1371/journal.pone.0308922)
Supplement: S7 File — Extended versions of Table 2 in the main paper. (DOCX) [file pone.0308922.s009.docx]

**Extended tables**

Extended versions of Table 2 in the main paper are provided below in Tables A, B, and C. Each row provides evaluation metrics for a different combination of input biomarkers, which shows the progression of building the best ensemble model by adding one input at a time. The best-performing model is shown in bold.

**Table A. Extended version of Table 2 in the main paper.** Each row provides evaluation metrics for a different combination of input biomarkers, which shows the progression of building the best ensemble model by adding one input at a time. The best-performing model is shown in bold.

**Table B. Extended version of Table 2 in the main paper.** Each row provides evaluation metrics for a different combination of input biomarkers, which shows the progression of building the best ensemble model by adding one input at a time. The best-performing model is shown in bold.

**Table C. Extended version of Table 2 in the main paper.** Each row provides evaluation metrics for a different combination of input biomarkers, which shows the progression of building the best ensemble model by adding one input at a time. The best-performing model is shown in bold.
